# Mechanically Robust Hybrid POSS Thermoplastic Polyurethanes with Enhanced Surface Hydrophobicity

**DOI:** 10.3390/polym11020373

**Published:** 2019-02-20

**Authors:** Xiuhuan Song, Xiaoxiao Zhang, Tianduo Li, Zibiao Li, Hong Chi

**Affiliations:** 1 Shandong Provincial Key Laboratory of Molecular Engineering, School of Chemistry and Pharmaceutical Engineering, Qilu University of Technology (Shandong Academy of Sciences), Jinan 250353, China; 18366102716@163.com (X.S.); zhang11071111@163.com (X.Z.); ylpt6296@vip.163.com (T.L.); 2 Institute of Materials Research and Engineering, A*STAR (Agency for Science, Technology and Research), 2 Fusionopolis Way, Innovis, #08-03, Singapore 138634, Singapore

**Keywords:** bi-functional POSS, hydrophobic modification, thermoplastic polyurethane, mechanical performance

## Abstract

A series of hybrid thermoplastic polyurethanes (PUs) were synthesized from bi-functional polyhedral oligomeric silsesquioxane (B-POSS) and polycaprolactone (PCL) using 1,6-hexamethylene diisocyanate (HDI) as a coupling agent for the first time. The newly synthesized hybrid materials were fully characterized in terms of structure, morphology, thermal and mechanical performance, as well as their toughening effect toward polyesters. Thermal gravimeter analysis (TGA) and differential scanning calorimetry (DSC) showed enhanced thermal stability by 76 °C higher in decomposition temperature (T_d_) of the POSS PUs, and 22 °C higher glass transition temperature (T_g_) when compared with control PU without POSS. Static contact angle results showed a significant increment of 49.8° and 53.4° for the respective surface hydrophobicity and lipophilicity measurements. More importantly, both storage modulus (G’) and loss modulus (G’’) are improved in the hybrid POSS PUs and these parameters can be further adjusted by varying POSS content in the copolymer. As a biodegradable hybrid filler, the as-synthesized POSS PUs also demonstrated a remarkable effect in toughening commercial polyesters, indicating a simple yet useful strategy in developing high-performance polyester for advanced biomedical applications.

## 1. Introduction

Polyurethane (PU) is a kind of thermoplastic elastomeric polymer, consisting of both hard and soft segments. The soft segments are usually composed of flexible polyester or polyether and the hard segments are usually composed of diisocyanates with benzyl structure [1]. Such alternating structure endows PU with good shape recovery property as well as high resistance to abrasion, stretchability [2], high adhesiveness and eases of processability because the hard phase can form a force center which holds the soft phase and retains the original shape [3]. Therefore, PU is widely applied in many fields including coating, foaming, adhesive, tissue engineering and holds a unique importance in daily life [4]. Despite the advantages as mentioned above, PU does show several drawbacks including low mechanical strength and short shelf-life. PU exhibits hydroscopic tendencies due to the hydrophilic urethane group. The poor hydrophobicity of polyurethane products could shorten their shelf-life as they absorb water and gradually decompose and lose their performance. Efforts have been devoted to improve the mechanical strength and hydrophobicity of PU by introducing various components to obtain organic-inorganic hybrid materials. The fillers can be graphene [5,6] small molecules [7], inorganic nanoparticles [8,9] and carbon nanotubes etc. [10]. For example, H. Jerry Qi and co-workers prepared photo-curable PUs made of aliphatic urethane diacrylate. Nanoparticles, such as fumed silica (200–300 nm), were added as a rheology modifier that imparts a shear thinning effect to uncured ink. Such a composition of the ink allows good printability as well thermal stability after printing [11]. Rigoberto C Advincula and coworkers used graphene oxide (GO) to further tailor their properties of polyurethanes and found that the addition of GO could enhance the mechanical property and thermal stability of PUs [12].

In the past few decades, polyhedral oligomeric silsesquioxanes (POSS) were reported to be a rising star in hybrid materials [13,14]. POSS represents the smallest molecular silica with the dimension in the range of 1–3 nm [15], and the general chemical formula of (RSiO_1.5_)_n_ (n= 6, 8, 12, etc.), where R could be a hydrogen atom/organic group, such as alkyl, aryl, vinyl, acrylate, epoxide group, to name a few [16]. The first merit to incorporate POSS into polymers chains is the super-hydrophobic tendency of POSS which mainly results from the surrounding R group [17,18,19,20]. The second merit is the biocompatibility and non-toxicity of POSSs [21,22,23,24]. Moreover, according to our previous results, not only could POSS enhance thermal stability [25,26], but it could also strengthen the mechanical properties [27] and retain the optical stability of the materials [28,29] due to its special composition and cage-liked nanostructure. In addition, POSS can be introduced into a polymer matrix by using covalently bonding as a pendent side group [30,31,32,33] or as end groups [34,35]. For example, Mya KY et al. [36] and Kun Wei et al. [37] reported star-shape POSS grafted PUs, which exhibited enhanced thermal stability and mechanical properties. Turri et al. [38,39] and Choudhury et al. [40] studied the surface properties of linear PUs which were modified with mono-functional POSS. It was found that the PU/POSS nanocomposites could significantly reduce the surface free energy and improve the surface hydrophobicity of the hybrids. In these ways, the properties of the polymer were less affected because the main chains of the polymers remain unchanged.

Herein, a series of hybrid PUs with POSS in the main chains were designed and synthesized. The obtained PUs showed enhanced thermal stability and mechanical property due to the existence of bi-functional POSS. Moreover, PCL was used as chain extender due to its excellent flexibility of the polymer chains [41]. Nuclear magnetic resonance (^1^H-NMR), fourier transform infrared spectroscopy (FTIR) and gel permeation chromatography (GPC) verified the successful synthesis of PUs. Static contact angle tests showed that the respective hydrophobicity and lipophilicity were remarkably increased by 49.8° and 53.4° compared with the results obtained on neat PU. Scanning electron microscopy (SEM) showed that there are microspores on the surface of PU films. Thermogravimetric analysis (TGA) and differential scanning calorimetry (DSC) indicated 76 °C higher decomposition temperature (T_d_) and 22 °C higher glass transition temperature (T_g_) compared to neat PU. Rheology tests suggested that both storage modulus (G’) and loss modulus (G’’) are improved in the hybrid PUs with the increasing ratio of POSS.

## 2. Materials and Methods

### 2.1. Materials

Phenyltrimethoxysilane (98%), methyldichlorosilane (97%), allyloxytrimethylsilane (98%), ε-caprolactone (99%) (ε-CL), Karstedt catalyst tin (II) 2-ethythexanoate (Sn(Oct)_2_) (96%) were all obtained from Alfa Aesar (Shanghai, China). Isopropyl alcohol, sodium hydroxide, triethylamine, dichloromethane, hexamethylene diisocyanate (HDI), polyethylene glycol (PEG: *M*_w_ = 1500) were purchased from Aladdin Reagent (Shanghai, China). PLGA (*M*_w_ = 100,000) was purchased from Sigma-Aldrich (Shanghai, China). All other solvents were purchased from Sino-pharm Chemical Reagent Co. Ltd. (Shanghai, China). Toluene, tetrahydrofuran and methanol were distilled before use, while other chemicals were used without further purification.

### 2.2. Synthesis of 3,13-dihydrooctaphenyl B-POSS

3,13-dihydrooctaphenyl B-POSS was synthesized as per our previous report [29]. Phenyltrimethoxysilane (15 mL, 0.08 mol), isopropyl alcohol (80 mL), deionized water (1.7 g, 0.09 mol) and sodium hydroxide (2.13 g, 0.05 mol) were charged to a three-necked flask under N_2_ atmosphere to synthesize octaphenyldicycloocatasiloxane tetrasodium silanolate Na_4_O_14_Si_8_(C_6_H_5_)_8_. The reaction was allowed to reflux at 96 °C for 4 h. After cooling down to room temperature, the mixture was stirred for additional 15 h. Solvent was removed via rotary evaporation and white powder was obtained after drying at 60 °C for 12 h in a vacuum oven. 3,13-dihydrooctaphenyl B-POSS was then obtained through the reaction between Na_4_O_14_Si_8_(C_6_H_5_)_8_ and methyldichlorosilane. Typically, a mixture of Na_4_O_14_Si_8_(C_6_H_5_)_8_ (11.24 g, 9.7 mmol), triethylamine (2.92 mL, 28.8 mmol) and anhydrous tetrahydrofuran (100 mL) in a flask was cooled down in an ice-water bath before the addition of methyldichlorosilane (3.385 g, 28.8 mmol) in tetrahydrofuran (10 mL). The reaction was conducted at 0 °C for one hour and 70 °C for another 3 h under dry nitrogen. White powder was collected via rotary evaporation and washed with 100 mL methanol three times. Finally, 4.57 g product with yield of 41 wt % was obtained. ^1^H-NMR (δ ppm, CDCl_3_): 0.38 (d, 6H, CH_3_–Si), 4.98 (d, 2H, Si–H), 7.14–7.50 (m, 40H, protons of aromatic rings).

### 2.3. Synthesis of 3,13-di(trimethylsilyl)oxypropyloctaphenyl B-POSS

3,13-di(trimethylsilyl)oxypropyloctaphenyl B-POSS was synthesized as per Wei et al.’s study [37]. 3,13-di(trimethylsilyl)oxypropyloctaphenyl B-POSS was synthesized via the hydrosilylation reaction between 3,13-dihydrooctaphenyl B-POSS and allyloxytrimethylsilane. Typically, 3,13-dihydrooctaphenyl B-POSS (5.44 g, 4.7 mmol), anhydrous toluene (50 mL), allyloxytrimethylsilane (3.67 g, 28.2 mmol) and the Karstedt catalyst were charged to a flask and the reaction was carried out at 95 °C for 36 h with vigorous stirring. Then, 6.76 g product with a yield of 82 wt % was collected after removing the solvent via rotary evaporation. ^1^H-NMR (δ ppm, CDCl_3_): 0.00 [s, 9H, –CH_2_CH_2_CH_2_OSi(CH_3_)_3_], 0.30 [s, 3H, –OSiCH_3_CH_2_CH_2_CH_2_OSi(CH_3_)_3_], 0.71 [t, 2H, –OSiCH_3_CH_2_CH_2_CH_2_OSi(CH_3_)_3_], 1.62 [m, 2H, –OSiCH_3_CH_2_CH_2_– CH_2_OSi(CH_3_)_3_] and 3.44 [t, 2H, –OSiCH_3_CH_2_CH_2_CH_2_OSi(CH_3_)_3_].

### 2.4. Synthesis of 3,13-dihydroxypropyloctaphenyl B-POSS

3,13-dihydroxypropyloctaphenyl B-POSS (B-POSS) was synthesized as per Wei et al.’s study [37]. 3,13-dihydroxypropyloctaphenyl B-POSS (B-POSS) was obtained through the deprotection reaction of 3,13-di(trimethylsilyl)oxypropyloctaphenyl B-POSS. 3,13-di(trimethlylsilyl)oxypropyloctaphenyl B-POSS (3.0 g, 2.12 mmol), anhydrous methanol (90 mL) and anhydrous CH_2_Cl_2_ (90 mL) in a flask were stirred and purged with nitrogen. Methyltrichlorosilane (0.68 g, 6.26 mmol) in anhydrous methanol (10 mL) was added dropwise to the solution within 30 min. The reaction was carried out at room temperature for 5 h, and then solvents were removed by rotary evaporation. Finally, 2.25 g powder was collected with yield of 93 wt % through washing the product with a mixture of THF and hexane (50/50 vol/vol) and dried in a vacuum oven. ^1^H-NMR (δ ppm, CDCl_3_): 0.31 (s, 3H, –OSiCH_3_CH_2_CH_2_CH_2_OH), 0.74 [t, 2H, –OSiCH_3_CH_2_CH_2_CH_2_OH], 1.63 [m, 2H,–OSiCH_3_CH_2_CH_2_–CH_2_OH], 3.47 [t, 2H, OSiCH_3_CH_2_CH_2_CH_2_OH]; MALDI-TOFMASS (product + Na+): 1292.1 Da (calculated: 1292.36 Da).

### 2.5. Synthesis of O-4000, O-8000, O-10000, O-12000 and O-14000

**Figure 1:**
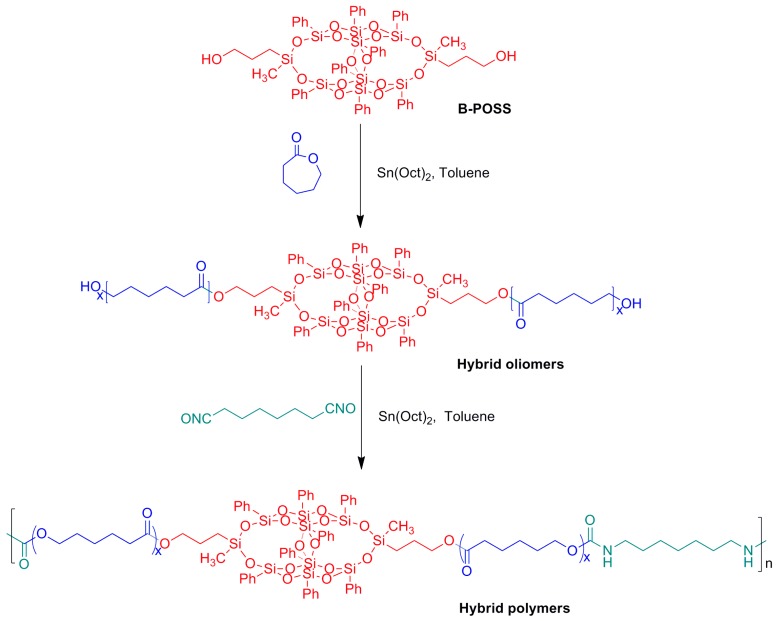
Synthesis of organic–inorganic hybrid PUs with B-POSS in the main chains.

The B-POSS incorporated oligomers of polycarprolactone (PCL) were synthesized with B-POSS and ε-CL by varying ε-CL content as shown in Figure 1. In a typical reaction, a three-necked round bottom flask charged with B-POSS (1.5 g, 1.18 mmol), ε-CL (8.07 g, 70.79 mmol), Sn(Oct)_2_ (375 μL) and anhydrous toluene (150 mL), vigorously stirred at room temperature for 5 min and then reacted at 110 °C for another 20 h under dry nitrogen. After that, the solvent was eliminated via rotary evaporation and the mixture was precipitated by adding an excessive amount of methanol. Then, the precipitate was obtained through centrifugation and washed with methanol three times. The product was dried in a vacuum oven at 40 °C for 24 h and then 8.13 g product of O-8000 was obtained with a yield of 85%. ^1^H-NMR (δ ppm, CDCl_3_): 1.4–1.6 (m, 6H –CH_2_CH_2_CH_2_–), 2.3 (t, 2H –COOCH_2_–), 4.1 (t, 2H –CH_2_OH–), 7.14–7.50 (m, 5H aromatic protons). FTIR (KBr, cm^−1^): 1536 (–NH–), 1730 (–COO–). GPC (PS standard, THF): *M*_w_ = 10,100, *M*_w_/M_n_ = 1.35. O-4000, O-10000, O-12000 and O-14000 were prepared using the same manner, simply varying the ε-CL feed content.

### 2.6. Synthesis of PUs (PU, PU-4000, PU-8000, PU-10000, PU-12000 and PU-14000)

To synthesize the designed linear PUs, the POSS-PCL oligomers are further reacted with HDI through stoichiometric and temperature control to produce five types of PUs with various physical properties as shown in Figure 1. In a typical procedure, O-8000 (6.5 g, 0.81 mmol) was dissolved in toluene and refluxed at 110 °C for 4 h. After cooling to 70 °C, Sn(Oct)_2_ (0.5 wt %, relative to the reactant) was added into solution and HDI (0.27 g, 1.62 mmol) in anhydrous toluene (10 mL) was added dropwise to the solution. The reaction was allowed to proceed at 70 °C for another 3 h under nitrogen. After that, the solvent was removed by rotary evaporation and PU-8000 was collected by precipitation with a large amount of ethyl ether. Finally, the product was obtained after drying at 40 °C for 24 h in a vacuum oven, and 5.65 g product was obtained with a yield of 83%. PU-4000, PU-10000, PU-12000 and PU-14000 were prepared through the same synthetic route with yields of 84%, 81%, 85% and 86%, respectively. Neat PU was obtained using PCL and HDI in the same way.

All the polyurethanes with B-POSS in the main chains were subjected to gel permeation chromatography (GPC) to measure their molecular weights. In all cases, high-molecular-weight products were obtained. It is noted that the polydispersity index (*M*_w_/M_n_) for these hybrid polyurethanes was slightly higher than that of the control polyurethane. The increased values of the polydispersity index could be attributed to the high steric hindrance of B-POSS. It is possible that the nanoscale size of B-POSS restricted the motion of the POSS macromer during the polymerization and that this effect was increasingly pronounced while its concentration was sufficiently high.

**Figure 2:**
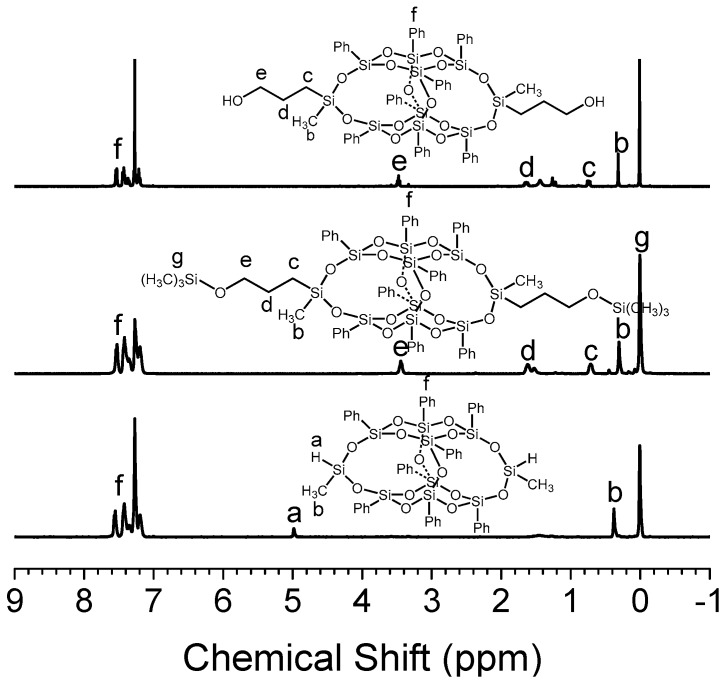
^1^H-NMR spectra of 3,13-dihydroxypropyloctaphenyl B-POSS (B-POSS), 3,13-di(trimethylsilyl) oxypropyloctaphenyl B-POSS and 3,13-dihydrooctaphenyl B-POSS from top to bottom.

The synthetic route of B-POSS-PUs is shown in Figure 2. To adjust the content ratio of B-POSS incorporated into the main chain of PU, different amounts of ε-CL were used as chain extender and grafted onto B-POSS, and the products were denoted as O-xxx to differentiate the molecular weight of polycaprolatone (PCL), i.e., O-4000 represents the PCL grafts with *M*_w_ of 4000 g/mol. 

### 2.7. Fabrication of Poly(lactic-co-glycolic acid) (PLGA) Composite Films

The as synthesized B-POSS, oligomers and PUs can be used as filler and doped into PLGA matrix by film casting with chloroform as a solvent. PLGA and B-POSS were dissolved in 100 mL of chloroform at room temperature. The mixture of the polymer and nanoparticles was placed in a Teflon dish, and the solution was evaporated at room temperature for 24 h. The formed film was vacuum-dried at room temperature for 24 h. The PLGA/B-POSS film was removed from the Teflon dish using liquid nitrogen. The film thickness was measured using a micrometer. PLGA/O-14000 and PLGA/PU-14000 were prepared through the same synthetic route.

## 3. Characterization

^1^H-NMR spectra were carried out on a Bruker AVANCE II 400 spectrometer (Bruker, Karlsruhe, Germany) using tetramethylsilane (TMS; d = 0 ppm) as the internal standard. Chemical shifts were reported in parts per million (ppm) and CDCl_3_ was used as solvent for all the samples.

Matrix-assisted ultraviolet laser desorption/ionization time-of-flight mass spectroscopy (MALDI-TOF-MS) (ultrafleXtreme MALDI-TOF/TOF, Bruker, Karlsruhe, Germany) with gentisic acid (2,5-dihydroxybenzoic acid, DHB) as the matrix and THF as the solvent was used to measure the molecular weight of B-POSS.

FTIR spectra were tested at room temperature via an IR Prestige-21 spectrometer (Bruker, Karlsruhe, Germany) operated at a resolution of 4 cm^−1^ with scan number of 32.

Thermogravimetric analysis was carried out using a thermogravimetric analyzer (TGA, SDT Q 600, TA Instruments, New Castle, DE, USA) at room temperature to 800 ° C at 10 °C/min under an air atmosphere.

The thermal behavior test of the polymer was measured using a DSC (Q10) differential scanning calorimeter (TA Instruments, New Castle, DE, USA). The polymer was heated from room temperature to 150 °C at a heating rate of 15 °C/min under air atmosphere.

The molecular weight and molecular weight distribution of the polymer were determined using THF as the eluent and polystyrene as the standard gel permeation chromatography (GPC, Shimadzu, Kyoto, Japan).

Scanning electron microscopy was used to characterize the surface morphology using SEM (QUANTA 200, FEI, Eindhoven, the Netherlands).

The rheological measurements of the polymer were carried out using a TA DHR-2 rheometer (TA Instruments, New Castle, DE, USA) on parallel plates of 20 mm diameter.

The tensile test was carried out using a microcomputer-controlled electronic universal testing machine (Hensgrand, WDW-02, Jinan, China) at a crosshead speed of 10 mm/min^−1^ and 25 °C.

## 4. Results and Discussion

### 4.1. Characterization of B-POSS

In this work, B-POSS was synthesized (Figure S1) and introduced into the main chains of PUs. ^1^H-NMR spectra of 3,13-dihydroxypropyloctaphenyl B-POSS (B-POSS), 3,13-di(trimethylsilyl) oxypropyloctaphenyl B-POSS and 3,13-dihydrooctaphenyl B-POSS is shown in Figure 2. The peaks of 3,13-dihydrooctaphenyl B-POSS appeared at 0.38 ppm (b), 3.41 ppm (e) and 7–8 ppm (f) were assigned to the protons of –Si–CH_3_, –CH_2_–OH and phenyl groups, respectively. The integration area ratio between b, e and f is 3.01: 2.04: 20.03, which is close to the theoretical ratios calculated from the structural formula. In Figure 2, the signals of 3,13-di(trimethylsilyl)oxypropyloctaphenyl B-POSS appeared at 0.38 ppm (b), 0.71 ppm (c), 1.62 ppm (d), 3.44 ppm (e) and 7–8 ppm (f), assigned to the protons of –Si–CH_3_, –CH_2,_ –CH_2_O and benzene rings, respectively. In addition, the peaks of Si–H protons at 4.98 ppm disappeared completely, indicating the successful hydrosilylation reaction. For B-POSS, the methyl protons peak of trimethylsilyloxypropyl at 0.02 ppm also completely disappeared after the deprotection reaction of 3,13-di(trimethylsilyl)oxypropyloctaphenyl B-POSS. Moreover, the successful synthesis of B-POSS was further confirmed by MALDI-TOF mass spectroscopy, where the molecular weight of B-POSS was determined to be 1292.1 (B-POSS + Na^+^), which is in good agreement with the calculated value (1269.1) from the molecular structure (Figure S2).

### 4.2. Characterization of the B-POSS-PUs

**Figure 3:**
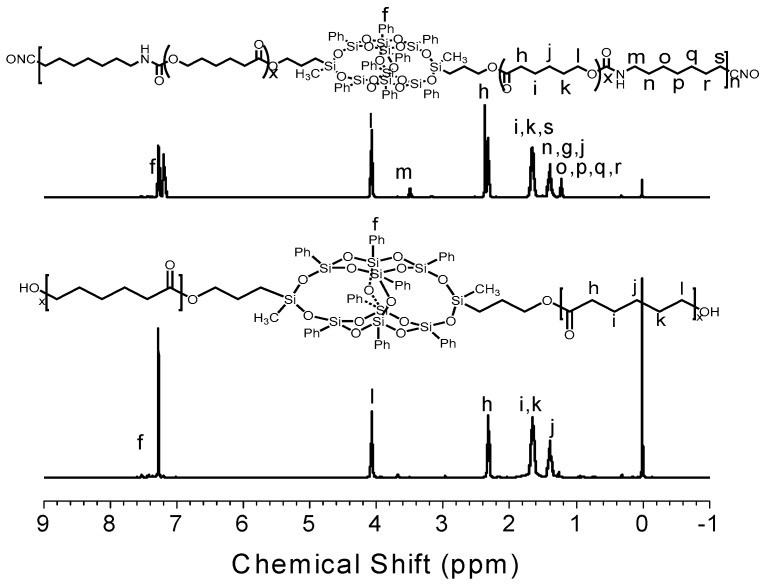
^1^H-NMR spectra of O-8000 and PU-8000 in CDCl_3_.

The typical ^1^H-NMR spectra of O-8000 and PU-8000 were presented as an example for the structure analysis of the as-synthesized PUs. As shown in Figure 3, the chemical shifts at 4.1 ppm (l), 2.3 ppm (h), 1.6 ppm (I, k), 1.4 ppm (j) were assigned to the proton of CH_2_ on PCL in O-8000, indicating that PCL was grafted onto B-POSS successfully. As for PU-8000, the chemical shifts at 3.5 (m), 1.2 ppm (o, p, q, r) was assigned to the protons of CH_2_ in the coupling regent HDI. The phenyl groups of B-POSS moieties were detected at 7.14–7.50 ppm in the spectrum, suggesting that B-POSS have been successfully incorporated into the main chain of PUs.

Through FTIR, the characteristic peak of –NCO in HDI at 2268 cm^−1^ disappeared after chain extension through urethane reaction (Figure S3, ESI). In addition, the appearance of strong bands at 1536 cm^−1^ and 1730 cm^−1^ assigned to –NH and –COO vibrations in the newly formed urethane linkage indicated the successful of reaction of POSS-PCL with HDI. ^1^H-NMR, FTIR together with GPC indicated the successful synthesis of organic–inorganic hybrid PU copolymers.

### 4.3. Thermal Stability

**Figure 4:**
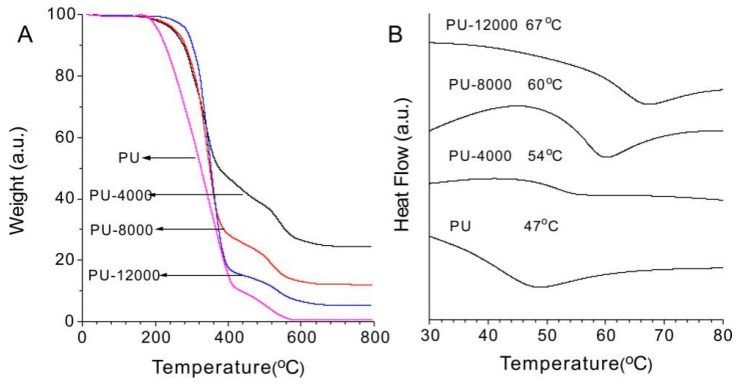
TGA curves of PU, PU-4000, PU-8000 and PU-12000 (**A**); DSC thermograms of PU, PU-4000, PU-8000 and PU-12000 (**B**).

Thermal stability and transitions of polymers were characterized by TGA and DSC analyses. TGA curves of PU, PU-4000, PU-8000 and PU-12000 are shown in Figure 4. One can see that the degradation temperatures (T_d_) are determined as 207, 238, 246, 266, 280 and 283 °C for neat PU, PU-4000, PU-8000, PU-10000, PU-12000 and PU-14000, respectively (Table 1). According to the molecular structure shown in Figure 1, different amount of ε-CL were reacted with B-POSS to adjust the chain length of PCL as well as the ratio of B-POSS in hybrid PUs. Therefore, the number of B-POSS on each polymer chain could be estimated based on the feeding ratio of ε-CL and the *M*_w_ of hybrid PUs, which was in the order of PU-14000 > PU-12000 > PU-10000 > PU-8000 >PU-4000 > PU, indicating that the thermal stability could be improved by B-POSS. The addition of POSS was reported to have significant effect in retarding the random breakage of the polymer backbone as well [38]. Comparison of the length of PCL in the hybrids with similar ratio of B-POSS indicated that PCL may also help to enhance thermal stability of the PUs because T_d_ also depends on the molecular weight (M_n_) of the polymer drastically [36], and the long-chain oligomers with POSS could enhances the thermal stability of the polyurethane by better dispersion [25,26,27,29]. The copolymer and neat PU exhibited similar TGA profiles, indicating that the incorporation of B-POSS had no significant effect on the degradation mechanism of the PUs backbone. The oxidation and decomposition of organic segments in pure PU and hybrid PUs are two stages of degradation. The residues in hybrid PUs were higher than those of neat PU which is probably due to the inorganic components in POSS. The formation of silica from thermal degradation of POSS is responsible for the residues after TGA run as reported from the FTIR analysis by Blanco and co-workers [42]. The silica coated on the surface of the material would protect the inner material from further decomposing due to its low thermal conductivity. Moreover, the weight percentage of B-POSS was in the order of PU-4000 > PU-8000 > PU-12000 > PU according to the molecular structure. Therefore, the decomposition yield of the residues could further confirm the better stability of the PUs due to the role of the B-POSS.

DSC curves of PU, PU-4000, PU-8000 and PU-12000 displayed in Figure 4B indicated that the glass transition temperatures (T_g_) are 47, 54, 60, 64, 67 and 69 °C for PU, PU-4000, PU-8000, PU-10000, PU-12000 and PU-14000 (Table 1), respectively. Compared with neat PU, hybrid PUs showed enhanced T_g_. This is probably due to rigid structure of POSS cages which could restrict the movement of the chain, thereby reducing the free volume; hence, a higher temperatures is required to provide the thermal energy to induce a glass transition in the polymers [43].

### 4.4. Surface Properties

**Figure 5:**
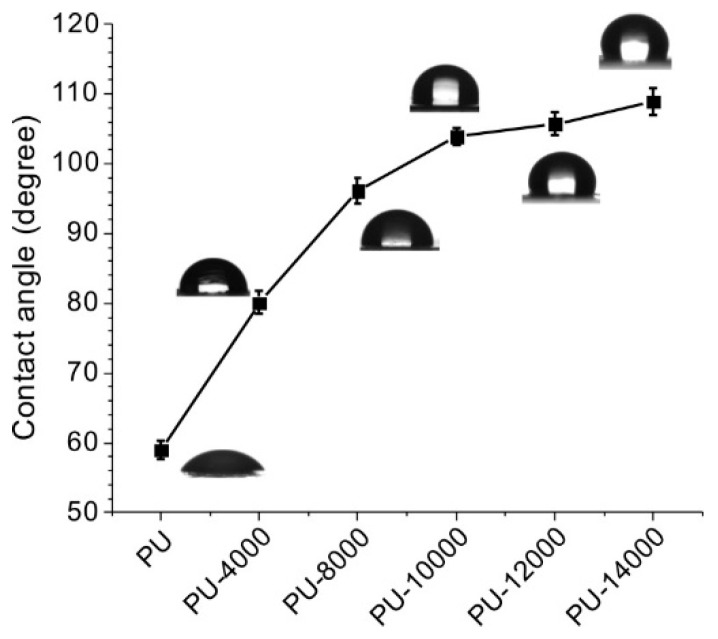
Plot of surface water contact angles of the organic–inorganic hybrid PUs.

Surface hydrophobicity of the hybrid PUs was investigated by static contact angle test. It has been reported that the incorporation of POSS cages can be used to enhance the hydrophobicity of polymers [27]. The hybrid PU polymers we synthesized in this study also exhibited hydrophobic feature as well as low surface free energy (Table 2). Static contact angles were tested with both water and ethylene glycol as probe liquids. Compared with neat PU, the water contact angle and ethylene glycol contact angles of the hybrid PUs were obviously improved. For example, the water contact angle of PU-14000 was as high as 108.9° (Figure 5), suggesting that the hydrophobicity and lipophilicity of PUs could be adjusted effectively by varying the B-POSS content in the copolymers.

The surface free energies of the organic-inorganic hybrid PUs were calculated according to the geometric mean model [44]:

cosθ = 2/γ_L_ [(γ^d^_L_ × γ^d^_s_)^1/2^ + (γ^p^_L_ × γ^p^_s_)^1/2^] (1)

γ_s_ = γ^d^_s_ + γ^p^_s_ (2)

where θ is the contact angle and γ_L_ is the liquid surface tension; γ^d^_L_ and γ^p^_L_ are the dispersive and polar components of γ_L_; γ_s_ is the solid surface tension, γ^d^_s_ and γ^p^_s_ are the dispersed and polar components of γ_s_ respectively. From this, the total surface free energies range of the organic-inorganic hybrid Pus are calculated to be in the range of 12.13 to 27.02 mN/m. The low surface energy endows the linear hybrids with potential application of highly hydrophobic coating materials. The presence of B-POSS cages could accumulate at the surface of the copolymers and thus minimize the polymer-air surface tension.

Surface morphology of the as-synthesized PUs were investigated by SEM. Figure S4 shows the SEM images of neat PU (A), PU-10000 (B), PU-12000 (C) and PU-14000 (D). Neat PU exhibited smooth surface morphology, whereas uniform pore distributions with the diameter of 1.08 μm were observed in PU-12000. Such an ordered self-assembly structure could be attributed to the phase separation between B-POSS and alkyl chains in PUs. This unique structural formation has been reported to be useful in lowering the dielectric constant value [45]. However, needle-like surface morphology was observed in PU-14000, which could be explained by the more effective separation of B-POSS from longer PCL chains. However, spindle-like surface morphology was observed in PU-14000, which could be explained by the longer chain of PCLs resulted packing.

### 4.5. Mechanical Properties

**Figure 6:**
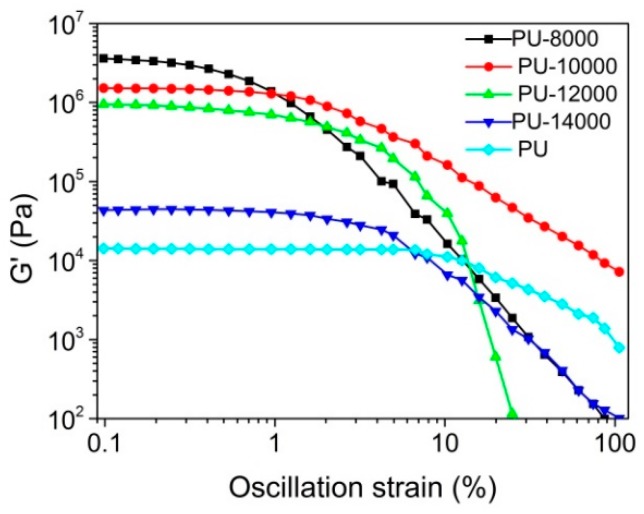
Dynamic strain sweep of: Gʹ for neat PU, PU-8000, PU-10000, PU-12000 and PU-14000.

The effect of different ratio of B-POSS on the mechanical property of PUs was investigated by measurement. A viscoelastic region was obtained through dynamic strain sweep test. As shown in Figure 6, the investigated region of storage modulus was viscoelastic and independent of strain. As the B-POSS content increased, the viscoelastic linear region in each sample becomes shorter because of the higher rigidity and brittleness of the sample as evidenced by the Payne effect [46]. Due to the disentanglement of polymer macromolecules, storage modulus (G’) decreases as strain increases in the slope region [47,48]. The presence of B-POSS may restrict the chain mobility of the PLGA. Dynamic frequency sweep was further studied, and the viscoelastic linear region of the materials was analyzed.

Dynamic frequency sweep was conducted at a strain of 0.5% to ensure the sufficient liner viscoelasticity region and sensitivity (Figure S5). G’ is higher than G’’ for all samples, suggesting that the samples are solid-like. Compared with neat PU, the hybrid PUs exhibited higher G’ and G’’ values and the increment is proportional to the POSS content in the copolymer. Rheology results indicated that the incorporation of B-POSS could improve the mechanical property and the mechanical strength of PU.

### 4.6. Influence on Mechanical Property of Poly(lactic-co-glycolic acid) (PLGA)

Due to the hybrid nature, the synthesized B-POSS, oligomers and hybrid PUs can be used as filler and doped into other polymer matrix to modulate their mechanical performances. To demonstrate the toughening effect of hybrid PUs, poly(lactic-co-glycolic acid) (PLGA) was selected as target polymer. As it is well-known, PLGA is a biodegradable functional polymer with good biocompatibility, non-toxicity and film-forming properties. It has been widely used in pharmaceutical, medical engineering materials and modern industrial fields [48]. However, the poor mechanical performance of PLGA has limited its application in more advanced areas. Interestingly, we found that the mechanical performance of PLGA could be greatly improved by blending with hybrid PUs in this study. The mechanical properties were investigated by tensile tests. The typical stress-strain curves in Figure S6 showed that the strength of the PLAG/POSS blends increased significantly whereas its high extensibility of PLGA was retained. After doping with 6.5 wt% of PU-14000, the mechanical strength was increased by 41.6% with a marginal sacrifice of the elongation-at-break (Table 3). This may result from the combined effects of high molecular weight of hybrid PUs and the good distribution of B-POSS in the blends. In addition, the B-POSS exhibited lowest toughness towards PLGA, probably because the rigid POSS materials could restrict the motion of PLGA chains and thus cause an increase in tensile strength and low strain. Among the tested samples, PLGA/O-14000 shows the highest toughness towards PLGA, as well as an improved elongation at breakage. Considering the use of organotins and diisocyanates precursors on the safety for pharmaceutical applications, Caracciolo, P.C. et al. [49] prepared a polyurethane elastomer network for drug transportation with DBTDL (dibutyltin dilaurate) as catalyst and diisocyanates as monomers. Gorna, K. et al. [50] reported using dibutyltin dilaurate as a catalyst and aliphatic hexamethylene diisocyanate, PCL and isosorbide glycol as precursors in preparing a biodegradable polyurethane scaffold for tissue repair and regeneration. Bonzani, I. C. et al. [51] also prepared a two-component injectable polyurethane for bone tissue engineering using pentaerythritol and glycolic acid 2,6-diisocyanate (ELDI) as a starting material under the catalysis of stannous octoate. Thus, such a simple yet useful strategy in modulating the mechanical performance of PLGA by hybrid PUs is promising in developing PLGA-based materials for more advanced biomedical applications.

## 5. Conclusions

In summary, a series of hybrid thermoplastic polyurethanes (PUs) incorporated with bi-functional polyhedral oligomeric silsesquioxane in the polymer backbone were successfully synthesized and fully characterized. With the introduction of B-POSS into the main chain, the surface hydrophobicity and thermal and mechanical stability of the hybrid PUs were significantly increased. The structure-property correlations were instigated by comparing with neat PU. As a new kind of thermoplastic filler, the hybrid PUs can also be used to improve the mechanical strength of PLGA, exhibiting promising application in biomedical devices.

## Figures and Tables

**Table 1:** Characteristics of PU, PU-2000, PU-4000 and PU-10000.

Polyurethane	*M*_w_ (g/mol)	M_n_ (g/mol)	*M*_w_/M_n_	T_d_ (°C)	T_g_ (°C)
PU	22,600	22,100	1.02	207	47
PU-4000	11,100	7930	1.39	238	54
PU-8000	23,100	15,100	1.53	246	60
PU-10000	29,900	19,200	1.56	266	66
PU-12000	36,400	22,700	1.60	280	67
PU-14000	45,200	27,100	1.67	283	69

**Table 2:** Static contact angles and surface free energy of PU and B-POSS.

	Static Contact Angle		Surface Free Energy (mN/m)		
Sample	Ө_H2O_	Ө_ethylene glycol_	γ^p^_s_	γ^d^_s_	γ_s_
PU	59.1 ± 0.6	40.2 ± 0.6	29.48	11.90	41.38
PU-4000	80.2 ± 0.8	63.2 ± 0.8	12.96	14.06	27.02
PU-8000	96.8 ± 0.9	77.1 ± 0.6	3.31	17.47	20.78
PU-10000	103.9 ± 0.6	86.8 ± 0.8	2.43	13.10	15.53
PU-12000	105.7 ± 0.8	88.9 ± 0.7	2.13	12.45	14.58
PU-14000	108.9 ± 0.9	93.6 ± 0.6	1.96	10.17	12.13

**Table 3:** Tensile strength and elongation at break of PLGA, PLGA/B-POSS, PLGA/O-14000 and PLGA/PU-14000.

Sample	Tensile Strength (MPa)	Elongation at Break (%)	Toughness (MJ/m^3^)
PLGA	0.36 ± 0.03	953.16 ± 22.3	241.85 ± 0.67
PLGA/B-POSS	0.44 ± 0.02	707.03 ± 14.2	229.10 ± 0.28
PLGA/O-14000	0.48 ± 0.01	833.62 ± 16.8	291.98 ± 0.17
PLGA/PU-14000	0.51 ± 0.03	725.52 ± 24.7	270.64 ± 0.74

## Supplementary Materials

The following are available online at http://www.mdpi.com/2073-4360/11/2/373/s1, Figures S1–S6: characterization of B-POSS, hybrid PUs.
