# Supplementary material for: Mechanically Robust Hybrid POSS Thermoplastic Polyurethanes with Enhanced Surface Hydrophobicity
Source: Polymers (Basel). 2019 Feb 20;11(2):373. doi: 10.3390/polym11020373 (PMC6419223; doi:10.3390/polym11020373)
Supplement: Supplementary file 1 [file polymers-11-00373-s001.pdf]

Mechanically Robust Hybrid POSS Thermoplastic Polyurethanes with Enhanced Surface Hydrophobicity

Xiuhuan Song [1], Xiaoxiao Zhang [1], Tianduo Li [1], Zibiao Li [2],* and Hong Chi [1],*

[1] Shandong Provincial Key Laboratory of Molecular Engineering, School of Chemistry and Pharmaceutical Engineering, Qilu University of Technology (Shandong Academy of Sciences), Jinan, China 250353

[2] Institute of Materials Research and Engineering, A*STAR (Agency for Science, Technology and Research), 2 Fusionopolis Way, Innovis, #08-03, Singapore 138634, Singapore

* Correspondence: ch9161@gmail.com; Tel.: +86-135-5319-7297

Received: 24 January 2019; Accepted: 18 February 2019; Published: 20 February 2019

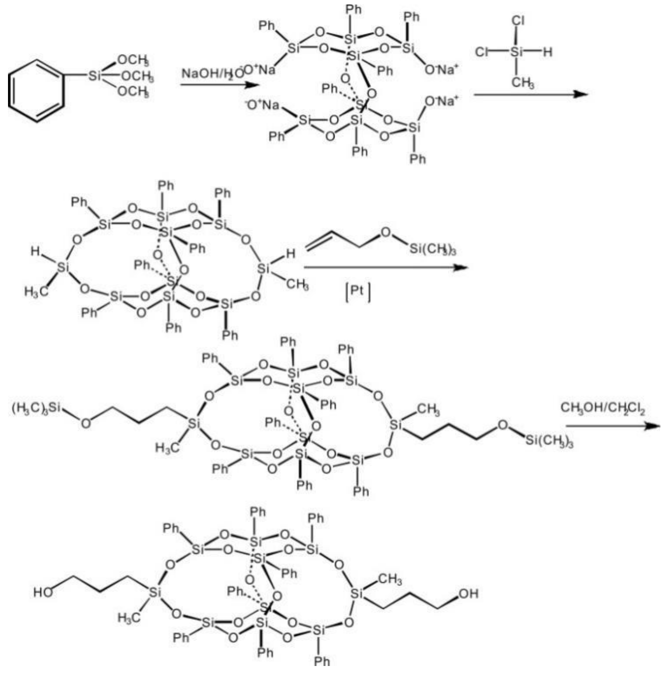

Figure S1. Schematic procedure of synthesize B-POSS.

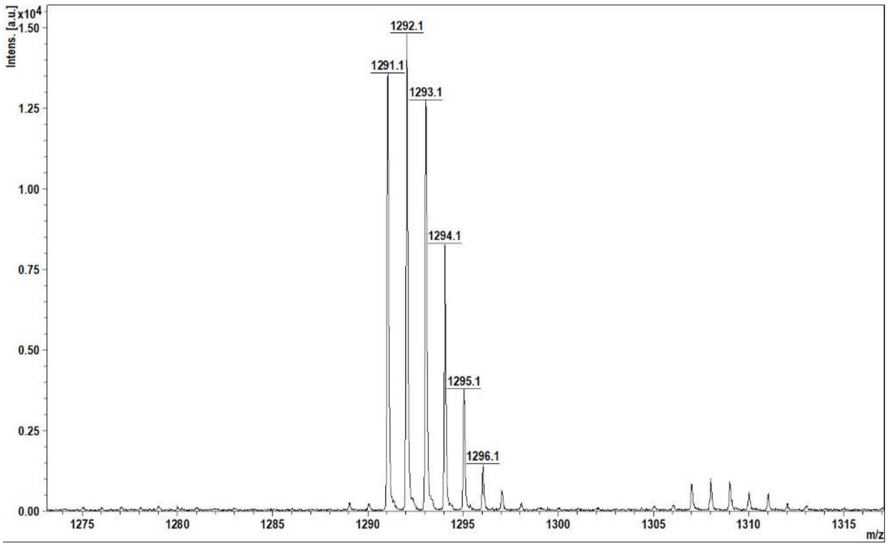

Figure S2. MALDI-TOF mass spectrum of B-POSS.

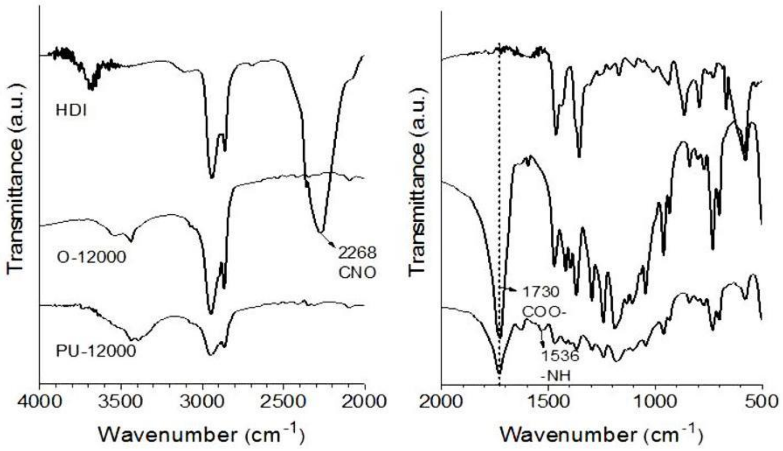

Figure S3. FTIR spectra of HDI, O-12000, PU-12000.

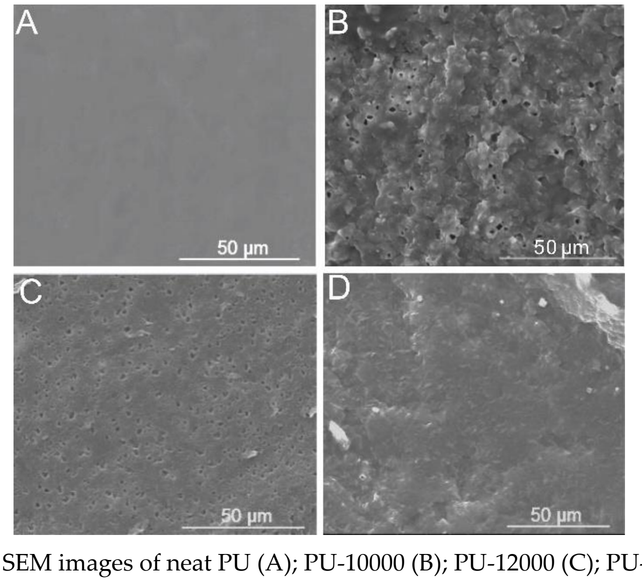

Figure S4. SEM images of neat PU (A); PU-10000 (B); PU-12000 (C); PU-14000 (D).

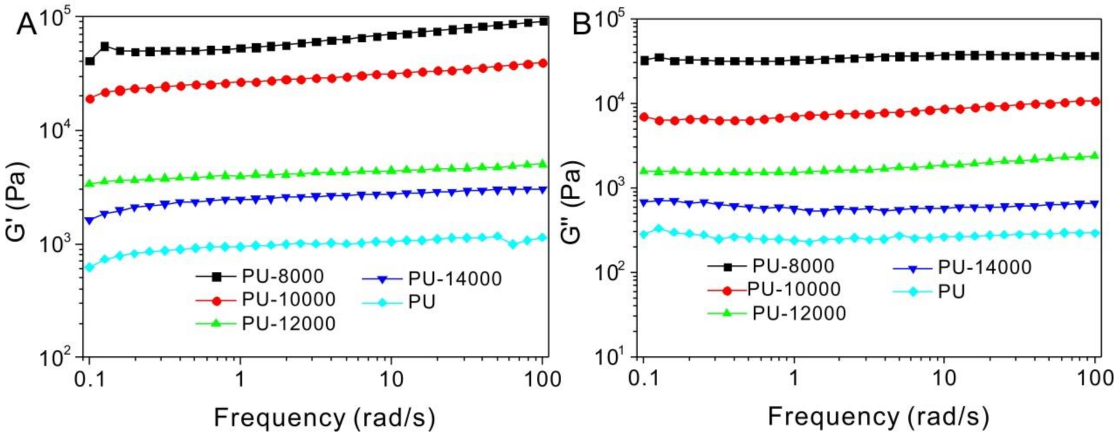

Figure S5. Dynamic frequency sweep of: G' (A) and G″ (B) for neat PU, PU-8000, PU-10000, PU-12000 and PU-14000 at 0.5% strain.

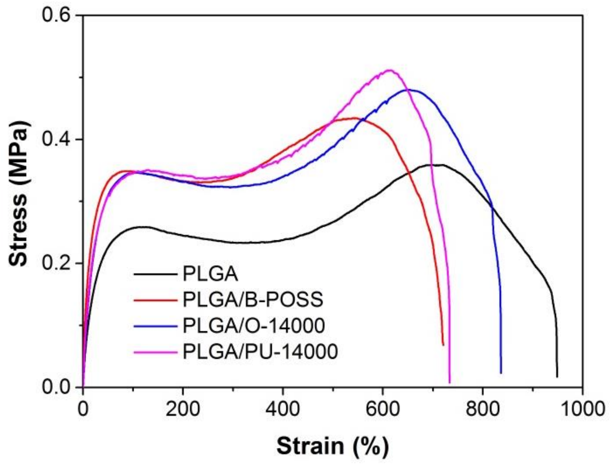

Figure S6. Stress–strain curves of PLGA, PLGA/B-POSS, PLGA/O-14000 and PLGA/PU-14000.
